# Interactions between Enterohemorrhagic Escherichia coli (EHEC) and Gut Commensals at the Interface of Human Colonoids

**DOI:** 10.1128/mbio.01321-22

**Published:** 2022-05-31

**Authors:** Fernando H. Martins, Anubama Rajan, Hannah E. Carter, Hamid R. Baniasadi, Anthony W. Maresso, Vanessa Sperandio

**Affiliations:** a Department of Microbiology, University of Texas Southwestern Medical Center, Dallas, Texas, USA; b Department of Biochemistry, University of Texas Southwestern Medical Center, Dallas, Texas, USA; c Department of Molecular Virology and Microbiology, Baylor College of Medicine, Houston, Texas, USA; New York University School of Medicine

**Keywords:** gut microbiota composition, EHEC, virulence regulation, human colonoids, exometabolome

## Abstract

The interactions between the gut microbiota and pathogens are complex and can determine the outcome of an infection. Enterohemorrhagic Escherichia coli (EHEC) is a major human enteric pathogen that colonizes the colon through attaching and effacing (AE) lesions and uses microbiota-derived molecules as cues to control its virulence. Different gut commensals can modulate EHEC virulence. However, the lack of an animal model that recapitulates the human pathophysiology of EHEC infection makes it challenging to investigate how variations in microbiota composition could affect host susceptibility to this pathogen. Here, we addressed these interactions building from simple to more complex *in vitro* systems, culminating with the use of the physiological relevant human colonoids as a model to study the interactions between EHEC and different gut commensals. We demonstrated that Bacteroides thetaiotaomicron and Enterococcus faecalis enhance virulence expression and AE lesion formation in cultured epithelial cells, as well as on the colonic epithelium, while commensal E. coli did not affect these phenotypes. Importantly, in the presence of these three commensals together, virulence and AE lesion are enhanced. Moreover, we identified specific changes in the metabolic landscape promoted by different members of the gut microbiota and showed that soluble factors released by E. faecalis can increase EHEC virulence gene expression. Our study highlights the importance of interspecies bacterial interactions and chemical exchange in the modulation of EHEC virulence.

## INTRODUCTION

Enteric pathogens must interact and outcompete a dense and diverse microbiota to establish a colonization niche in the gastrointestinal (GI) tract (1, 2). Microbiota-derived signaling molecules and metabolites can be used by pathogens as cues to control virulence gene expression and metabolism in the gut (3–6). It is well known that interindividual differences in microbiota composition can profoundly affect the host susceptibility to infection and disease severity, inasmuch as genetically identical murine lines, with natural occurring variations in their microbiota compositions, showed different colonization levels and disease susceptibility after Citrobacter rodentium infection, a murine enteric pathogen. Moreover, transfer of fecal microbiota from resistant to susceptible animals could confer resistance against the infection (7). Therefore, investigating the complex interactions between commensals and pathogens is crucial to understanding the progression of enteric infections.

Enterohemorrhagic Escherichia coli (EHEC) is a human foodborne pathogen that colonizes the colon and causes outbreaks of bloody diarrhea and hemolytic uremic syndrome (HUS) worldwide. The two main features of EHEC pathogenesis are the production of Shiga toxins, which are responsible for the clinical symptoms associated with HUS, and the ability to colonize the intestinal epithelium through formation of attaching and effacing (AE) lesions (8, 9). Most genes required for AE lesion formation are clustered within a pathogenicity island (PAI) named locus of enterocyte effacement (LEE), which encodes a type III secretion system (T3SS) that injects many effector proteins into the host cell, leading to cytoskeleton rearrangement and effacement of the intestinal microvilli (10). In addition, EHEC can produce mucin-degrading enzymes such as the zinc metalloprotease StcE, which contributes to bacterial penetration of the mucus layer toward the colonic epithelium (11).

The infectious dose of EHEC is remarkably low (12), highlighting the extremely efficient mechanisms evolved by this pathogen to expand in the gut. For instance, EHEC can take advantage of the resident gut commensals by using microbiota-derived molecules both as nutrients and as signals to regulate its virulence genes, including the LEE, since the T3SS expression is energetically costly and, therefore, tightly regulated (13, 14). The observation that infected individuals with the same strain of EHEC manifest varying degrees of disease severity, suggests that variations in the microbiome could influence host susceptibility to EHEC infection (15–17). Furthermore, previous studies have shown that different members of the gut microbiota can affect EHEC virulence and disease (18–20).

The lack of a suitable murine animal model that recapitulates the human pathophysiology of EHEC infection makes it difficult to investigate the mechanisms underlaying the modulation of EHEC virulence by gut microbes. The use of human-derived intestinal organoids has dramatically advanced the study of enteric bacterial pathogenesis and provides a powerful pluripotent platform to deepen our understanding of host–pathogen interactions (21–23). Intestinal organoids are composed of heterogeneous cell types and recapitulate many aspects of the normal human GI tract physiology (24).

Here, we used human intestinal organoids as a physiologically relevant model to explore the interactions between EHEC and gut commensals. In summary, we show that EHEC attachment to colonic epithelium is enhanced in the presence of Bacteroides thetaiotaomicron and Enterococcus faecalis, two phylogenetically distinct gut commensals, and is unaltered by commensal E. coli. Moreover, we identified several changes in metabolites by the presence of specific gut commensals and showed that soluble factors released by E. faecalis can increase EHEC virulence gene expression. This study highlights the importance of microbiota–pathogen interactions in the outcome of enteric infections.

## RESULTS

### Gut commensals modulate EHEC virulence.

**FIG 1:**
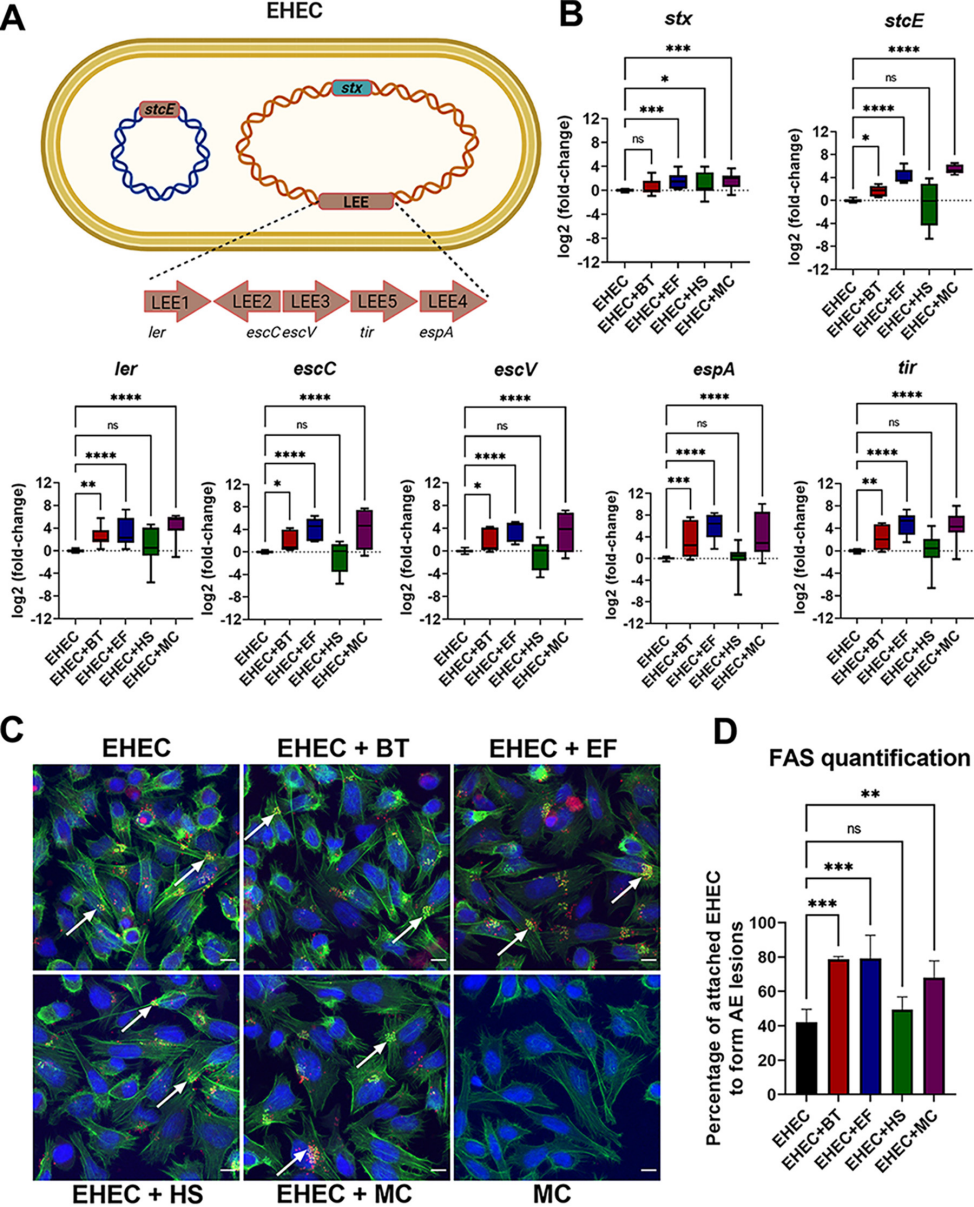
Bacteroides thetaiotaomicron and Enterococcus faecalis, but not commensal Escherichia coli, enhance EHEC virulence. (A) A schematic of the EHEC major virulence genes: locus of enterocyte effacement (LEE), *stx* (encoding for Shiga toxin), and *stcE* (encoding for StcE mucinase). (B) EHEC was anaerobically grown alone or in coculture with commensal strains, and transcription of virulence genes was measured by targeted qRT-PCR. (C) FAS assay to detect actin pedestal formation on HeLa cells infected with mCherry-expressing EHEC and commensal strains for 6 h. Actin and DNA were stained with FITC-phalloidin (green) and DAPI (blue), respectively. White arrows indicate sites of actin polymerization (pedestals). Original magnification: 63×. Scale bar: 10 μm. (D) Quantification of FAS showing the percentage of attached EHEC cells to form actin pedestals. The number of attached EHEC and actin pedestals were enumerated in multiple fields (*n* = 4). Error bars represent the means ± SD from at least three independent experiments. MC, mixed culture (*B. theta*, E. faecalis, and commensal E. coli). Statistical significance was determined by one-way ANOVA followed by a *post hoc* Tukey test. *, *P < *0.05; **, *P < *0.01; ***, *P < *0.001; ****, *P < *0.0001; ns, not significant.

Enteric pathogens encounter hundreds of different bacterial species in the gut, many of which can impact both their colonization and virulence gene expression (2, 5). To investigate how different gut microbiota bacteria impact EHEC virulence gene expression, we selected three phylogenetically diverse commensal bacteria for a series of coculture experiments. EHEC was grown alone or with one or more representative members of the three major gut microbiota phyla: Bacteroides thetaiotaomicron (BT; *Bacteroides* phylum), Enterococcus faecalis (EF; *Firmicutes* phylum), and commensal E. coli strain HS (HS; *Proteobacteria* phylum). Virulence gene expression was measured via quantitative PCR (qRT-PCR) (Fig. 1A and B). In agreement with previous reports (18, 19), both B. thetaiotaomicron (*B. theta*) and E. faecalis, but not E. coli HS, enhanced expression of EHEC LEE genes (*ler*, *escC*, *escV*, *tir*, and *espA*) (Fig. 1B). Regarding non-LEE-encoded genes, *B. theta* and E. faecalis also increased the expression of the mucinase-encoding gene *stcE*, while upregulation of *stx* was observed in coculture with E. faecalis and E. coli HS (Fig. 1B). We also observed upregulation of virulence gene expression when EHEC was cocultured with a mixed culture (MC) containing all three commensal strains, suggesting that the presence of commensal E. coli was not enough to inhibit the phenotype conferred by *B. theta* and E. faecalis (Fig. 1B).

A hallmark of EHEC infection is its ability to induce host actin remodeling at the sites of bacterial attachment, resulting in a pedestal-like structure (25). To enumerate actin pedestal formation, we infected HeLa cells with mCherry-expressing EHEC in the presence or absence of the gut commensals and performed a fluorescent actin staining (FAS) assay. Cells were stained with fluorescein isothiocyanate (FITC)-phalloidin, and pedestals were visualized as bright green puncta of polymerized actin beneath attached red bacteria. The average percentage of attached EHEC cells to form pedestals was significantly higher in the presence of *B. theta* and E. faecalis compared with the levels for EHEC alone or EHEC cocultured with E. coli HS (Fig. 1C and D). Congruent with the LEE expression data, pedestal formation was also enhanced in the presence of all three commensal strains (Fig. 1C and D).

**FIG 2:**
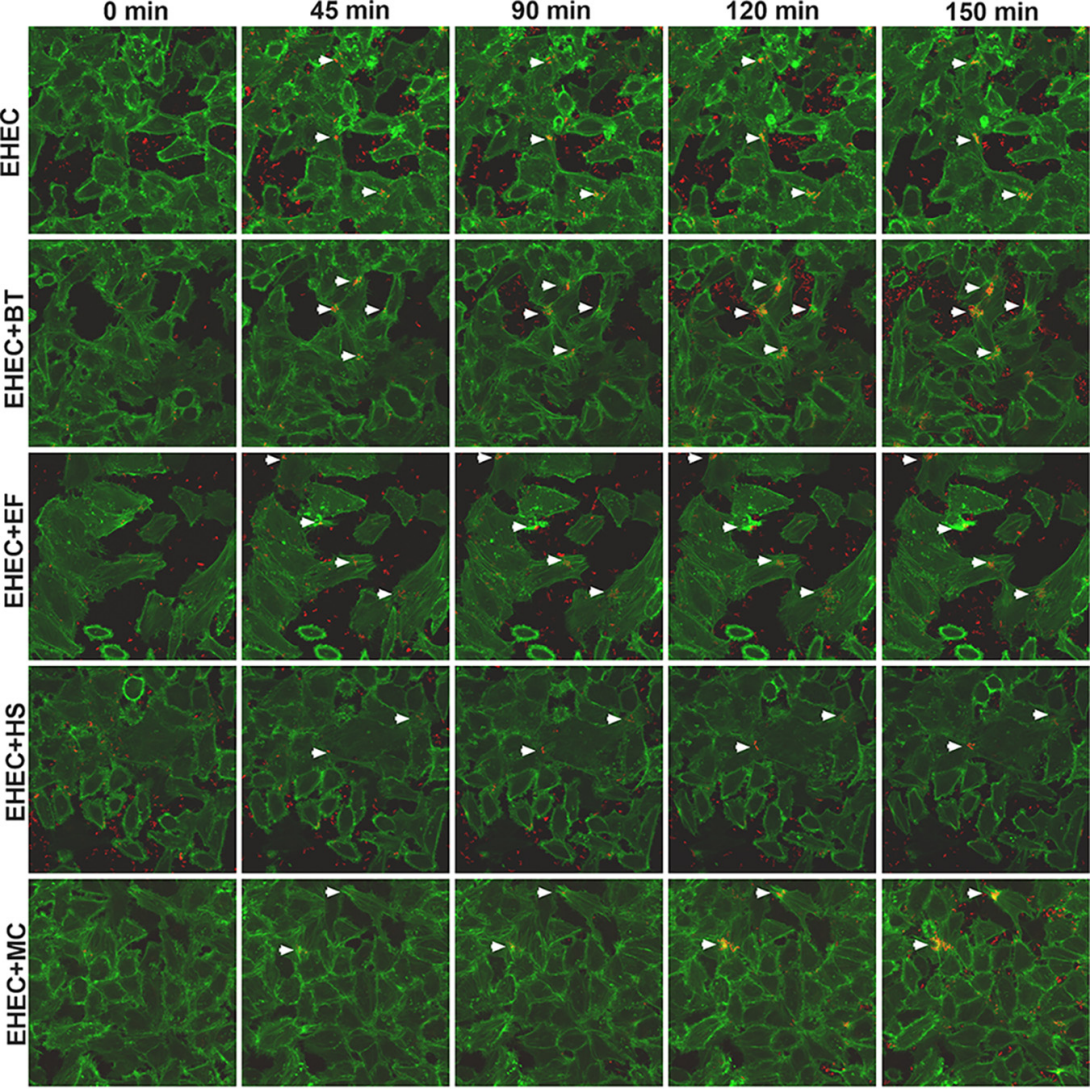
Time-lapse microscopy of Lifeact::GFP-expressing HeLa cells infected with mCherry-expressing EHEC and commensals strains. A representative field of each infection condition was imaged for 2.5 h, and images taken every 5 min. White arrowheads indicate clusters of pedestal-forming bacteria. Original magnification: 63×.

Next, we compared the dynamics of pedestal formation by live-cell imaging. Lifeact::GFP-expressing HeLa cells were infected with mCherry-expressing EHEC, and actin pedestal formation was evaluated at different time points of infection. Here again, we observed more pedestals being formed in the presence of *B. theta*, E. faecalis, and all three commensals (MC), but not E. coli HS (Fig. 2 and Movies S1–S5). However, we did not observe any significant change in the kinetics of AE lesion formation, suggesting that the actin pedestals were not formed faster, but only in higher numbers in the presence of *B. theta*, E. faecalis, or both together (Fig. 2 and Movies S1–S5). Overall, these results indicate that actin pedestals were more efficiently formed in the presence of *B. theta* and E. faecalis, congruent with the virulence gene expression data.

### EHEC attachment to colonic epithelium is enhanced by gut commensals.

**FIG 3:**
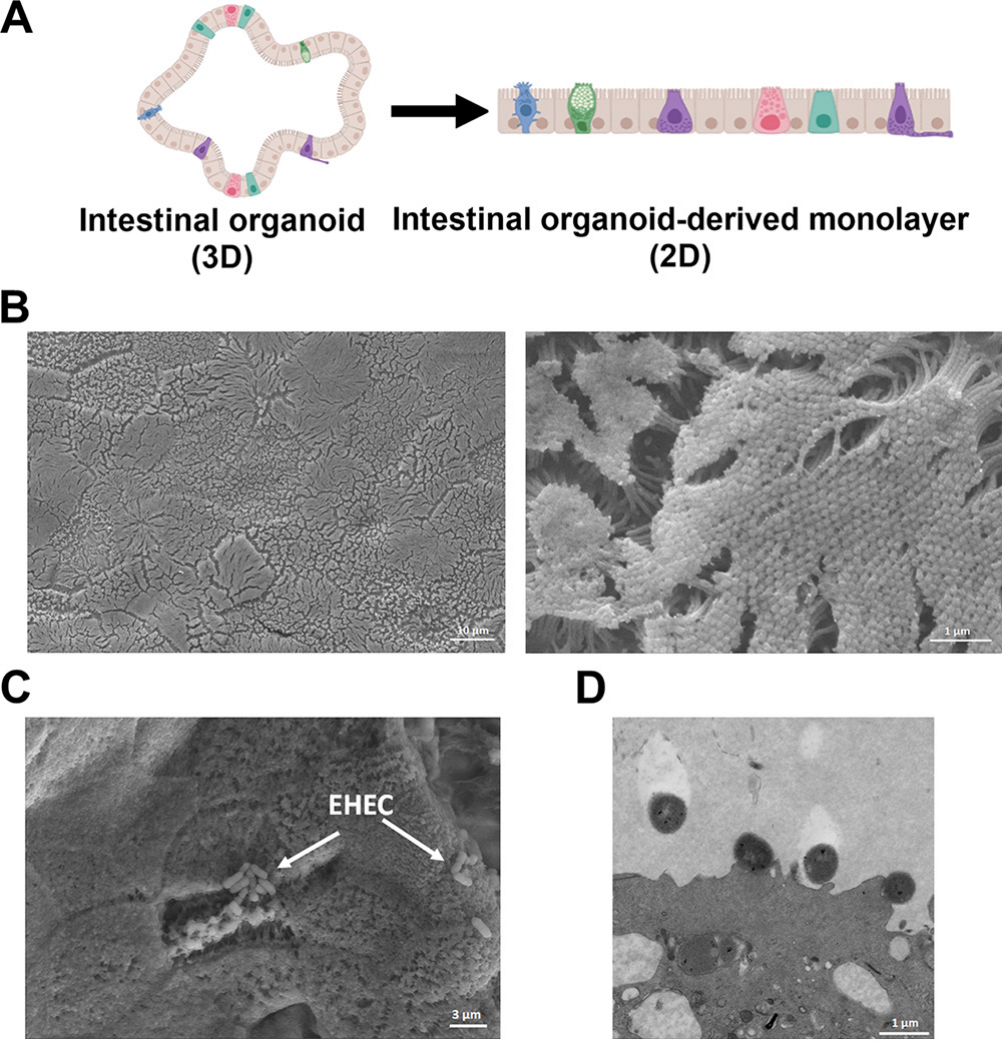
Human colonoids as a model for EHEC infection. (A) A schematic of 3D and 2D (monolayer) human colonoids. (B) Scanning electron microscope (SEM) images of uninfected colonoids (mock controls) showing the monolayer surface (low magnification, left panel) and highlighting the brush border and microvilli (high magnification, right panel). (C) SEM images of EHEC-infected colonoid monolayers after 6 h. White arrows point to clusters of attached EHEC. (D) Transmission electron microscope (TEM) images of infected colonoid monolayers showing the presence of actin pedestals formed by EHEC.

**FIG 4:**
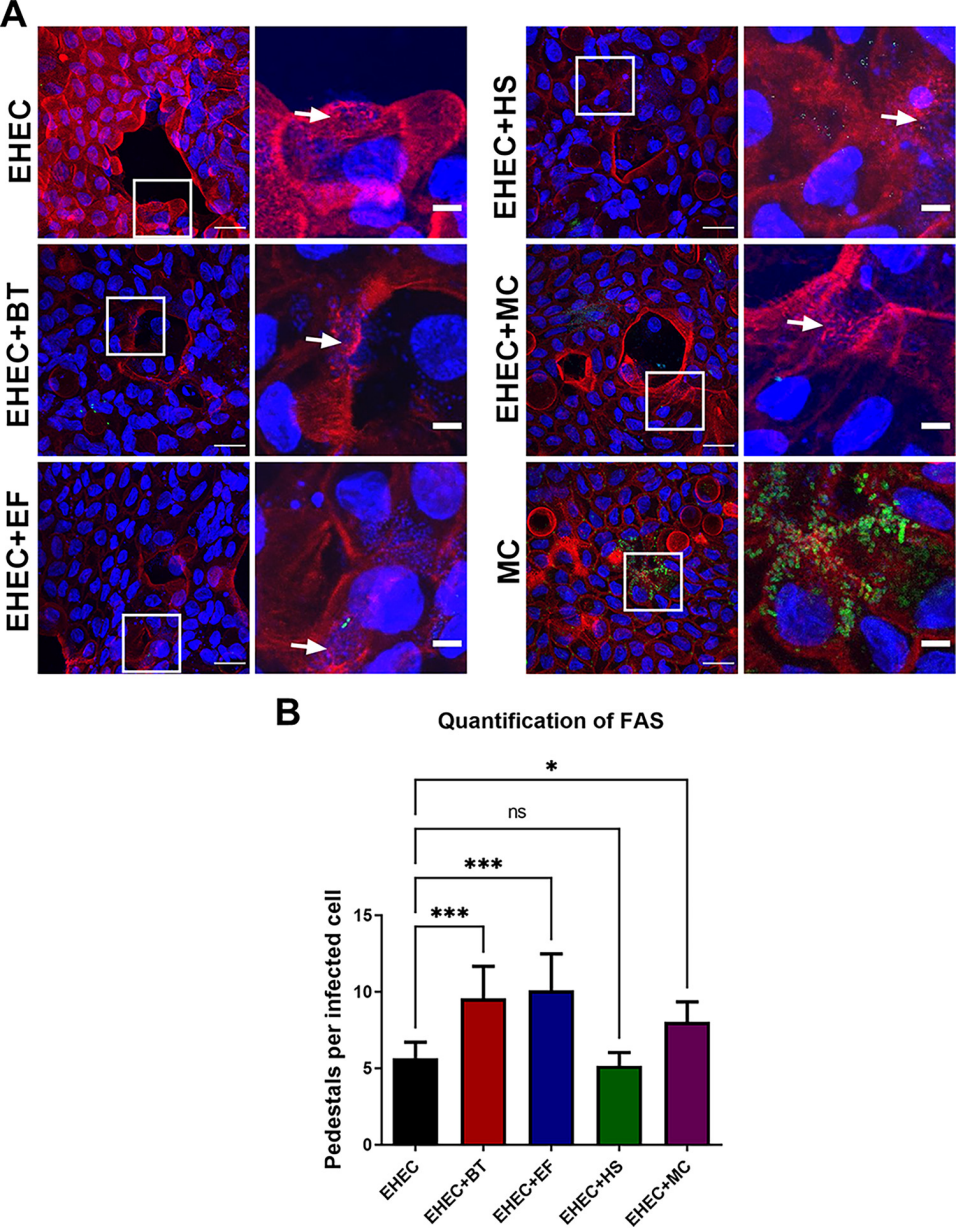
Gut commensals enhance EHEC attachment to human colonoids. (A) FAS assay to detect pedestal formation on colonoid monolayers infected with EHEC and GFP-expressing commensal strains for 6 h. Actin and EHEC/DNA were stained with Alexa Fluor 568-phalloidin (red) and DAPI (blue), respectively. White arrows indicate sites of EHEC attachment and actin pedestal formation. Original magnification: 63×. Scale bar: 10 μm (left panels), 5 μm (right panels). (B) Quantification of FAS showing the number of pedestals per infected cells enumerated in multiple fields (*n* = 4). Error bars represent the means ± SD, and statistical significance was determined by one-way ANOVA followed by a *post hoc* Tukey test. *, *P < *0.05; ***, *P < *0.001; ns, not significant.

HeLa cells are routinely used in the field to quantify pedestal formation by AE pathogens (26). However, it is evident that this model does not sufficiently represent the complex multicellular environment of the human intestine. To quantify actin pedestal formation in a more complex and physiologically relevant model, we infected monolayers derived from human colonoids (Fig. 3A). These colonoids are composed of all the major cell types of the intestinal epithelium (villus enterocytes, Paneth, goblet, enteroendocrine, and stem cells), contain a lumen, microvilli, and crypt, secrete mucin, and are physiologically active. Colonoids can be grown as 3-dimensional or 2-dimensional monolayers (Fig. 3B). We used 2-dimensional differentiated colonoids since EHEC preferentially attaches to them compared to undifferentiated ones, and they are easier to image, as previously described (27). EHEC attached and formed AE lesions on these colonoid monolayers as depicted through both scanning electron microscopy (Fig. 3C) and transmission electron microscopy (Fig. 3D). Using the fluorescence microscopy, we observed that more actin pedestals were formed by EHEC in the presence of *B. theta* and E. faecalis and the three members of the microbiota combined, but not with E. coli HS (Fig. 4A and B), recapitulating our previous findings on HeLa cells. Notably, GFP-expressing commensal strains, mostly E. coli, were also able to attach to the colonic epithelium (Fig. 4A).

### The presence of gut commensals does not significantly change the colonic transcriptional response to EHEC infection.

**FIG 5:**
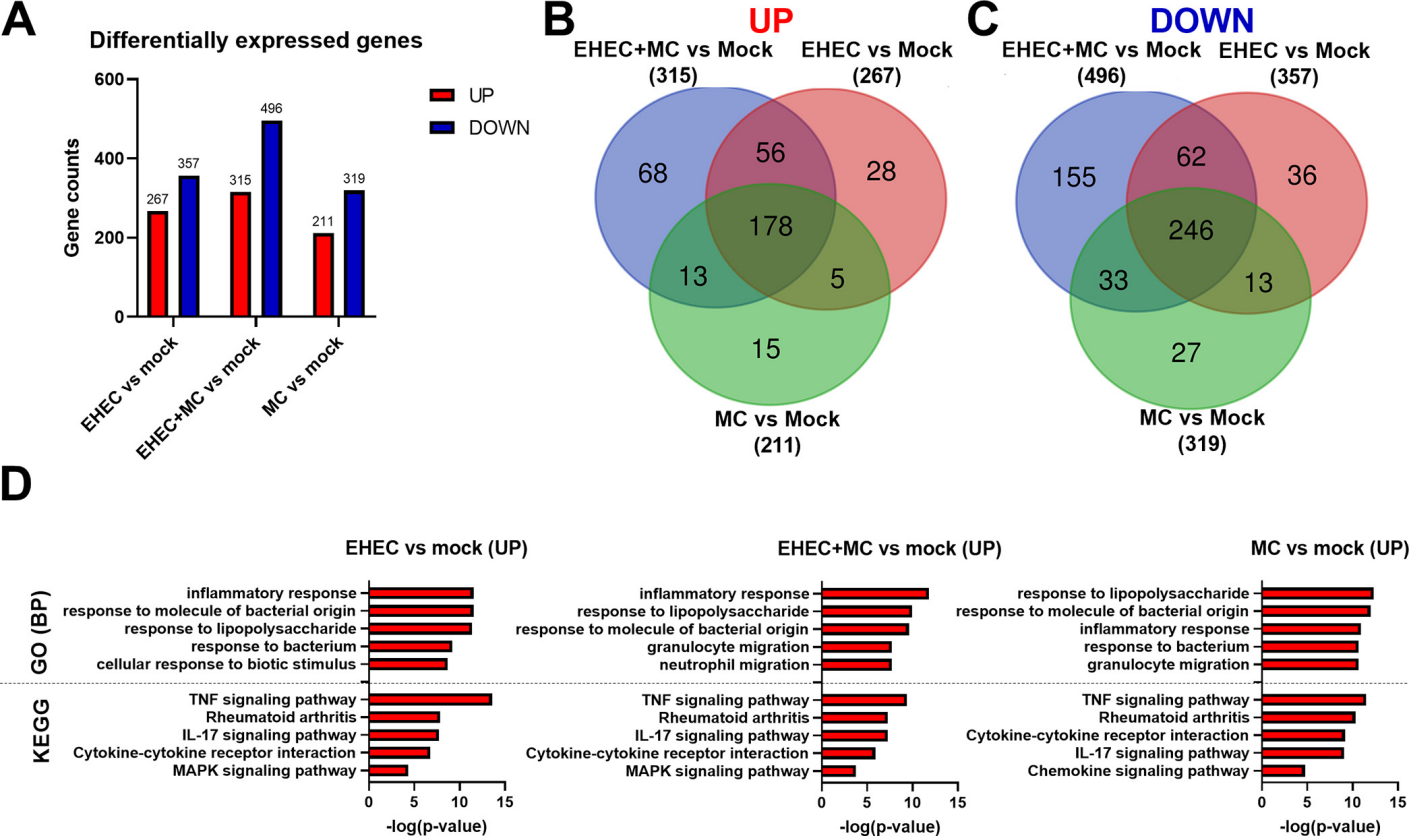
Colonic transcriptional response to infection with EHEC and commensal strains. Colonoid monolayers were infected for 6 h with EHEC, EHEC in the presence of commensals (EHEC+MC), or commensals only (MC), and transcriptomes were analyzed by RNA sequencing. (A) Total number of differentially expressed genes (DEGs) in infected colonoid monolayers versus mock controls. Red and blue indicate up- and downregulated genes, respectively. (B) Venn diagram showing the number of upregulated genes in each comparison (EHEC versus mock, EHEC+MC versus mock and MC versus mock). (C) Venn diagram showing the number of downregulated genes in each comparison. (D) Upregulated gene set enrichments showing the top 5 GO Biological Processes and KEGG pathways in infected colonoid monolayers over mock controls.

To gain insight into intestinal epithelium responses during EHEC infection and the relative contributions of the gut microbiota, we identified differentially expressed genes (DEGs) between mock infected samples and colonoid monolayers infected with EHEC, EHEC in the presence of all commensals (EHEC+MC), or commensals only (MC) (Table S5 at https://www.dropbox.com/scl/fi/4sc2x8k7hbi22vlm00ueq/Table-S3.xlsx?dl=0&rlkey=obe2yjtnw2k4kc2ggsgbgyigc and Fig. 5). We found higher numbers of DEGs in colonoid monolayers infected with EHEC in the presence of commensals compared to infections with EHEC or commensals only (Fig. 5A). Overall, a total of 234 and 308 genes were commonly up and downregulated, respectively, in response to EHEC infection, regardless of the presence or absence of commensal strains (Fig. 5B and C). Other 223, 64, and 42 DEGs were unique to colonoid monolayers infected with EHEC+MC, EHEC, or commensals only, respectively (Fig. 5B and C).

To identify biological processes and pathways associated with DEGs from each infection condition, gene sets were separated into upregulated (increased) and downregulated (decreased) categories based on fold change relative to mock infected samples and imported separately into the GO and KEGG databases (Fig. 5D). In the upregulated data sets, most genes were associated with GO Biological Processes terms commonly linked to bacterial infection, including response to molecule of bacterial origin, response to lipopolysaccharide, and inflammatory response (Fig. 5D and Table S3 in the supplemental material). Most significant enriched pathways in all three infection conditions belonged to the immune system category, including TNF and IL-17 signaling, Rheumatoid arthritis, and cytokine–cytokine receptor interaction (Fig. 5D and Table S3). To our surprise, the downregulated DEG data sets were not significantly associated with any GO Biological Process or KEGG pathway, nor were the DEGs unique to each condition. Taken together, these results revealed that inflammatory pathways were the primary responses during EHEC infection, regardless of the presence of the gut commensals. Notably, the infection of colonoids with commensal strains only also resulted in upregulation of genes related to inflammatory response.

### Gut commensals change the metabolic landscape.

**FIG 6:**
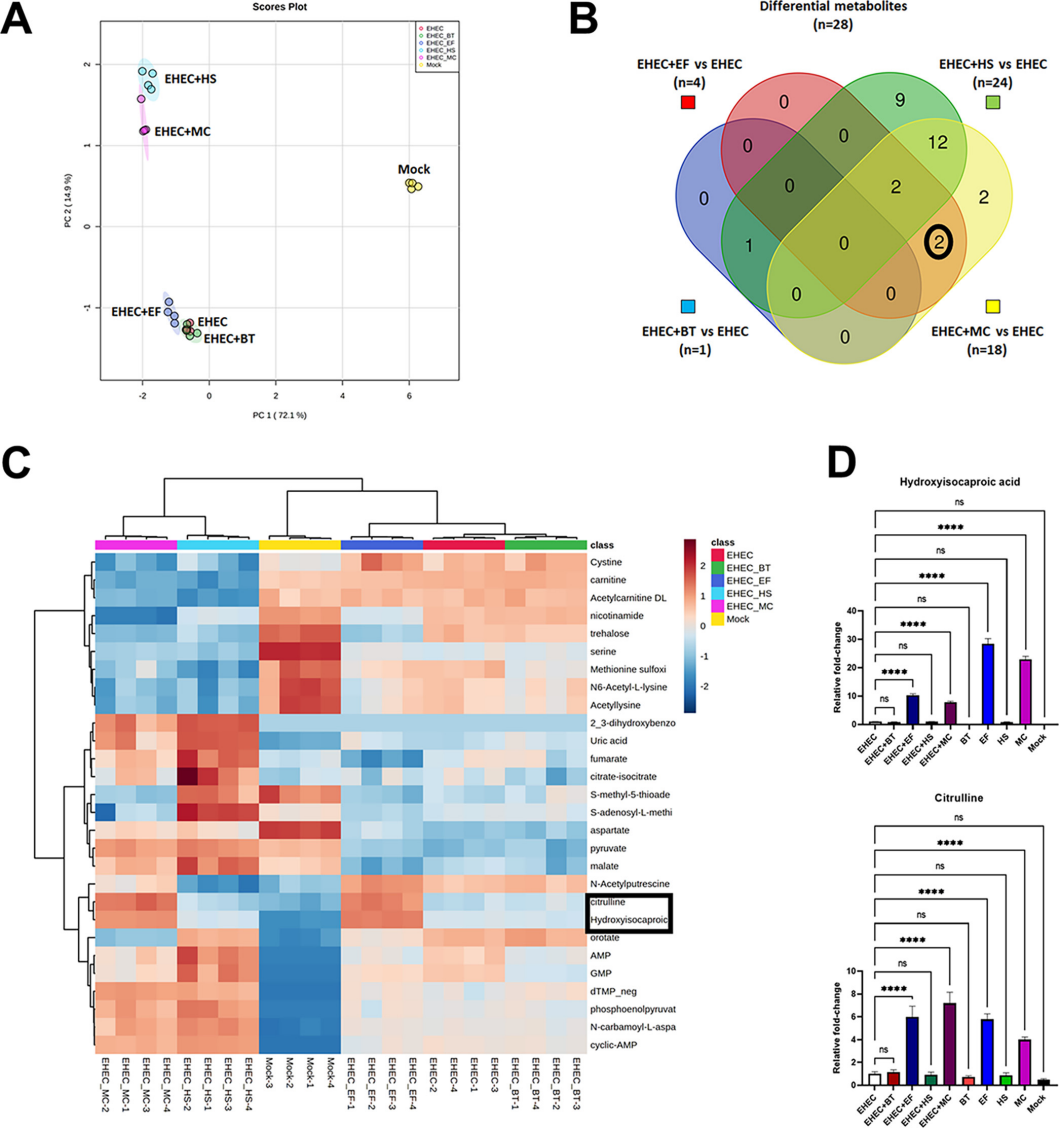
Gut commensals change the metabolic landscape. Colonoid monolayers were infected with EHEC and commensal strains for 6 h, and metabolites in the extracellular media (exometabolome) were identified by LC-MS/MS. (A) Principal-component analysis (PCA) of the profiling data from the colonoid monolayers exometabolome, showing clear separation among the groups (infected and mock controls). (B) Venn diagram illustrating metabolites that were differentially abundant (fold change > 2.0, FDR < 0.05) in coinfections with commensal strains compared to colonoid monolayers infected with EHEC alone. (C) Heatmap of the 28 differential metabolites showed in the Venn diagram. Red and blue indicate higher and lower levels, respectively. (D) Relative abundance of hydroxyisocaproic acid and citrulline (highlighted in both the heatmap and the Venn diagram) in the exometabolome of mock and infected colonoid monolayers compared to results with EHEC alone. Statistical significance was determined by one-way ANOVA followed by a *post hoc* Tukey test. ****, *P < *0.0001; ns, not significant.

EHEC controls its virulence program in response to both host and microbiota derived metabolites (3–6). To identify metabolic changes promoted by specific members of the gut microbiota, we analyzed and compared the exometabolomes (extracellular metabolites) of colonoid monolayers infected with EHEC alone or in the presence of the commensal strains. Principal-component analysis (PCA) revealed changes in the metabolic landscape promoted by the presence of gut commensals (Fig. 6A). Specifically, infections of colonoid monolayers with EHEC alone or in the presence of *B. theta* or E. faecalis resulted in a more similar metabolic landscape, while the exometabolomes of colonoids coinfected with EHEC and E. coli HS or all commensal strains were quite diverse (Fig. 6A).

We confidently identified 102 extracellular metabolites with differential abundance (Table S4), and 28 of them showed higher or lower abundance (differential metabolites) in the colonoids coinfected with EHEC and microbiota strains compared to the ones only infected with EHEC (Fig. 6B). Congruent with the PCA, a higher number of differentially abundant metabolites were identified in colonoids coinfected with EHEC and E. coli HS or all commensal strains, while only four, and one metabolite were changed in the exometabolomes of colonoid monolayers infected with E. faecalis and *B. theta*, respectively (Fig. 6B and C). Two metabolites were significantly increased in the presence of E. faecalis: hydroxyisocaproic acid and citrulline. These metabolites were also very abundant in the exometabolomes of colonoid monolayers infected with E. faecalis or all commensals together in the absence of EHEC (Fig. 6D).

### E. faecalis-derived signals increase LEE expression.

**FIG 7:**
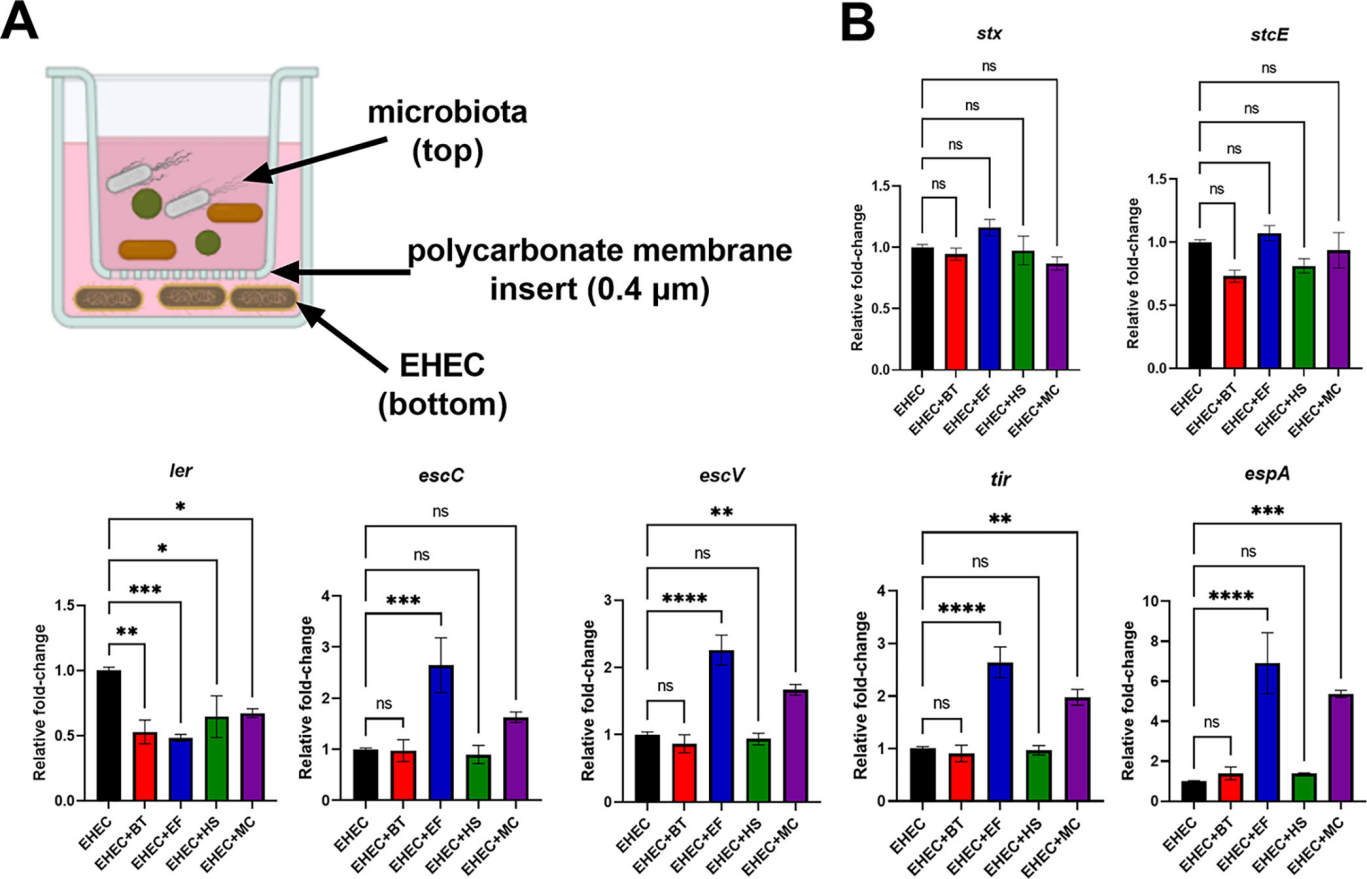
E. faecalis-derived soluble factors are sufficient to increase EHEC virulence gene expression. (A) A schematic of the transwell system. Gut commensals (top compartment) and EHEC (bottom compartment) are grown together while physically separated by a 0.4 μm pore polycarbonate membrane insert, which allows chemical communication between both bacterial populations. (B) Transcriptional levels of virulence genes were measured by targeted qRT-PCR. Error bars represent the means ± SD from two independent experiments (six biological replicates). Statistical significance was determined by one-way ANOVA followed by a *post hoc* Tukey test. *, *P < *0.05; **, *P < *0.01; ***, *P < *0.001; ****, *P < *0.0001; ns, not significant.

Next, we used a transwell system to test whether metabolic shifts promoted by gut commensals was sufficient to affect EHEC virulence in the absence of potential cell-to-cell contact. This model allows chemical exchange between bacteria in a contact-independent manner (Fig. 7A). By doing that, we observed that coculturing EHEC with E. faecalis, but not with *B. theta* or E. coli HS, resulted in upregulation of most LEE genes (Fig. 7B). We note that expression of the *ler* transcription factor was downregulated under these conditions in contrast to its upregulation when these strains were in contact with each other (Fig. 1B). Ler is the master transcription regulator of all LEE genes (13). However, there are several potential explanations for this phenotype. Transcription of *ler* occurs early during infection and is downregulated later compared to the other LEE operons (1). The fact that *ler* expression is upregulated in the coculture experiment could be due to a stronger stimulus when EHEC and commensals are in close proximity, which could keep LEE expression at high levels even after 6 h. In fact, the relative increase in LEE expression is higher in the coculture experiments compared to the transwell ones (Fig. 1 and 7). Moreover, we cannot exclude the possibility that other Ler independent and/or post-transcriptional mechanisms induced by the presence of commensals could be modulating the LEE expression. We note that there are many reports of downstream posttranscriptional regulation of the *LEE2–LEE5* operons (13).

Expression levels of the non-LEE genes *stx* and *stcE* were not affected, suggesting that the mechanism driving the upregulation of these genes require contact or closer proximity between EHEC and gut commensals. Similar results were obtained when EHEC was grown in the presence of all three microbiota species (Fig. 7B). Altogether, these findings indicate that soluble factors released by E. faecalis were sufficient to increase LEE expression, but not *stx* and *stcE*.

## DISCUSSION

Variations in microbiota composition are a critical factor in determining host susceptibility to enteric infections (7, 28). There is growing evidence that microbiota composition can also impact the course of EHEC-mediated disease, given that people infected with the same strain during an outbreak show a wide range of differential symptoms and disease progression (15, 17, 29). Here, we show that Bacteroides thetaiotaomicron and Enterococcus faecalis, two phylogenetically distinct commensal bacteria, increase EHEC virulence gene expression and attachment to epithelial cells (Fig. 1, 2, and 4). Our findings corroborate previous reports showing that these two gut microbes can enhance EHEC virulence (18, 19). Interestingly, a study investigating changes in the gut microbiota of patients with EHEC compared to healthy controls showed a higher abundance of E. faecalis in the infected group (16). Thus, it is tempting to speculate that these commensals could create a mutualistic relationship and benefit from EHEC infection, although further studies are still needed to elucidate that.

The use of human intestinal organoids has extended our understanding of many aspects related to host–pathogen interactions (21–23). Many classical features of EHEC pathogenesis have been confirmed in this more complex and physiologically relevant model, such as the ability to degrade mucus and form AE lesions in the colonic epithelium (27), and the damage promoted by Shiga toxin to the intestinal cells (30). To the best of our knowledge, this is the first study to use the human colonoids as a model to explore the interactions between EHEC and different members of the gut microbiota. We also observed enhanced EHEC virulence upon infection of colonoid monolayers in the presence of *B. theta* and/or E. faecalis (Fig. 4), corroborating our findings in the coculture experiments and infections of HeLa cells (Fig. 1 and 2).

EHEC colonization of the intestinal epithelium elicits a robust inflammatory response (31). By coculturing human intestinal organoids and polymorphonuclear cells, Karve et al. (32) showed an upregulation of inflammation markers that are key to neutrophil recruitment upon EHEC infection. Here, we investigated whether the presence of a multispecies gut microbial community could impact the colonic response to EHEC infection. Overall, we observed a strong upregulation of genes and pathways related to immune responses and inflammation in both infection conditions (Fig. 5). However, the infection of colonoid monolayers with only commensal strains also elicited inflammatory responses, making it difficult to attribute any role played by the microbiota in the colonic response to EHEC infection. Since we observed attachment of E. coli HS to the colonoids (Fig. 4A), we speculate that exposure to microbial antigens such as lipopolysaccharide (LPS) and flagellin would be exerting an inflammatory response in the intestinal epithelium, masking responses due to AE lesion formation and Shiga toxin production by EHEC in this colonoid model.

Metabolic shifts associated with changes in the microbiota composition can have significant effects on the outcome of enteric infections (7). EHEC can sense and regulate its virulence in response to different microbiota-derived metabolites, such as indole (33), succinate (18), fucose (34), and ethanolamine (35), among others. Also, a multi-omics study showed that metabolites derived from the human, but not the murine, microbiome were sufficient to increase EHEC damage to intestinal epithelial cells (36). Here, we demonstrate that the infection of colonoid monolayers with EHEC in the presence of different commensal bacteria resulted in differential metabolic landscape shifts, where the most significant changes were observed in coinfections with E. coli HS (Fig. 6). However, the metabolic shift promoted by commensal E. coli does not seem to be affecting EHEC virulence, since we did not observe an enhancement of bacterial attachment in this infection condition.

Proteases produced by *B. theta* and E. faecalis promote higher effector translocation, enhancing pedestal formation by EHEC (19, 37). However, the proteases activity does not explain the transcriptional changes induced by these commensals on EHEC. Indeed, a protease-deficient E. faecalis strain is still able to enhance pedestal formation by EHEC (19). Thus, we hypothesized that metabolic changes driven by *B. theta* and E. faecalis also affect EHEC virulence. Many metabolites change in the intestine of mice coinfected with *B. theta* and C. rodentium, another AE pathogen (18). However, we observed fewer changes in the metabolic profile promoted by *B. theta* in the exometabolome of the colonoid monolayers. These data suggest that there are differences between these two model systems, and that their limitations and complementarity must be acknowledged.

Co-culturing of EHEC with E. faecalis resulted in enhanced virulence in all different models used in this study, including the transwell system (Fig. 7). Previous studies have shown metabolic cross-feeding between E. faecalis and other bacteria, including E. coli (38). In our metabolomics study, two metabolites were more abundant and associated with the presence of E. faecalis: hydroxyisocaproic acid, a by-product of leucine catabolism (39), and citrulline, a precursor of arginine (40). Although we observed increased EHEC virulence in coinfections with E. faecalis, it is still unclear whether these metabolites can regulate EHEC virulence or are only biomarkers of E. faecalis metabolism.

Our findings show that specific members of the gut microbiota can promote changes in the intestinal microenvironment and differentially impact EHEC virulence. This work has fundamental implications for how differential microbiota compositions may affect disease outcome and susceptibility to enteric pathogens. Defining the mechanisms by which the microbiota affects the regulation and activity of virulence factors expressed by intestinal pathogens would represent a significant step forward in the field of microbiota-pathogen interactions.

## MATERIALS AND METHODS

### Bacterial strains, plasmids, growth, and cell culture conditions.

All bacterial strains and plasmids used in this study are listed in Table S1. EHEC and E. coli HS were routinely grown in Luria-Bertani (LB) medium. E. faecalis V583 was routinely grown in Brain Heart Infusion (BHI) medium. B. thetaiotaomicron was routinely grown in TYG medium (41). For coculture experiments, Dulbecco’s modified Eagle’s medium with 1 g/L glucose (low-glucose DMEM) was used, as these conditions are known to induce EHEC virulence gene expression (42). Anaerobic growth was performed using a GasPak EZ anaerobe container system (Becton, Dickinson). When adequate, media were supplemented with ampicillin (100 μg/mL), gentamicin (50 μg/mL), chloramphenicol (20 μg/mL), erythromycin (10 μg/mL), or streptomycin (100 μg/mL). Bacterial stocks were kept on LB supplemented with glycerol 30% (vol/vol) at −80°C.

HeLa (human cervical adenocarcinoma) cells were maintained in DMEM with 4.5 g/L glucose (high-glucose DMEM) supplemented with 10% fetal bovine serum (FBS) and penicillin-streptomycin-glutamine. The Lifeact::GFP-expressing HeLa cell line was obtained with the Flip-In system (Invitrogen) as previously described (43), and maintained in high-glucose DMEM supplemented with 10% FBS, penicillin-streptomycin-glutamine, and hygromycin (50 μg/mL). Hygromycin was not added when cells were split before infection. Cells were routinely grown at 37°C and 5% CO_2_.

### Coculture experiments.

To measure the expression of EHEC virulence genes in coculture experiments, we used a strain engineered to present a unique housekeeping gene, the chloramphenicol resistance gene (*cat*) expressed under the control of the EHEC *rpsM* promoter (19). This engineering was necessary because of the high sequence homology among genes commonly used as endogenous controls from EHEC and commensal E. coli, which makes impossible the specific amplification of housekeeping genes for normalization. Bacterial strains were grown anaerobically at 37°C overnight and spun down, and bacterial pellets were resuspended in low-glucose DMEM. Fresh medium was inoculated at 1:100 with the bacterial suspensions. *B. theta* was added at 10-fold excess over EHEC to mimic ratios in the gut, while E. faecalis and E. coli HS were added at 1:1 ratio. Cultures were grown anaerobically for 6 h at 37°C, and immediately placed on ice after the incubation. Bacterial cells were collected by centrifugation, resuspended in TRIzol (Invitrogen), and stored at −80°C.

For transwell experiments, bacteria were grown in a 12-well plate containing a 12 mm Transwell with 0.4 μm pore polycarbonate membrane insert (Corning). Commensal strains were added to the top compartment (volume: 0.5 mL), while EHEC was grown in the bottom (volume: 1 mL). Inoculum preparation, growth conditions, and sample processing were done as described above. Samples were collected from both compartments and plated onto selective media to assure that bacteria were not passing through the filters, resulting in a mixed culture.

### RNA isolation and qRT-PCR.

RNA was extracted from bacterial cultures using a RiboPure Bacteria isolation kit (Ambion) according to manufacturer’s protocols. A total of 2 μg of RNA was used for cDNA synthesis. qRT-PCR was performed using diluted cDNA samples, validated primers (Table S2), and SYBR green Mix in a Quantstudio 6 Flex Real-Time system (Applied Biosystems). Data were collected using QuantStudio Real-Time PCR Software v1.3, normalized to the expression levels of the engineered housekeeping gene *cat*, and analyzed using the comparative critical threshold (CT) method. A *P* of <0.05 was considered significant, and multiple independent coculture experiments were performed, with three replicates in each.

### Fluorescent actin staining (FAS) assay.

Fluorescent actin staining (FAS) assays were performed as described by Knutton et al. (25) to examine actin pedestal formation. Briefly, HeLa cells were grown on coverslips in 12-well plates to about 80% confluence. Cells were washed with phosphate-buffered saline (PBS), and fresh low-glucose DMEM supplemented with 10% FBS was added. Overnight bacterial cultures were resuspended in fresh low-glucose DMEM and used to infect the cells at a multiplicity of infection (MOI) of 100 for each bacterial strain, except for *B. theta* (MOI of 1,000). All strains were simultaneously added to the cells, and infections were allowed to proceed for 6 h at 37°C and 5% CO_2_. After 3 h, the cells were washed with PBS to remove nonattached bacteria and the medium was replaced. After infection, the cells were washed again, fixed with formaldehyde and permeabilized with 0.2% Triton X-100. The preparations were then treated with fluorescein isothiocyanate (FITC)-labeled phalloidin and 4’,6-diamidino-2-phenylindole (DAPI) to visualize actin accumulation and bacteria/HeLa DNA, respectively. Slides were mounted with ProLong antifade (Molecular Probes) and visualized with a Zeiss LSM880 confocal laser scanning microscope. Pedestal formation was quantified by randomly imaging different fields of view while recording the number of cells showing actin accumulation foci. At least four fields were enumerated for each condition, with each field containing at least 20 cells. The number of attached EHEC cells was also enumerated in each field. All strains were tested in duplicates, and at least three independent experiments were performed.

### Live cell-imaging.

Lifeact::GFP-expressing HeLa cells were cultured in 35-mm glass-bottom dishes (MatTek) and then infected with mCherry-expressing EHEC, in the presence or absence of commensals, as described above. Infections were allowed to continue for 2 h at 37°C and 5% CO_2_; the cells were then washed and visualized by live-cell imaging with an Olympus Fluoview Fv10i confocal laser scanning microscope. Images were taken every 5 min for 2.5 h.

### Human colonoid culture.

The human colonoid line TC202 was purchased from the Baylor College of Medicine GEMs enteroid core. Three-dimensional (3D) colonoids were cultured in Matrigel (Corning), and colonoid monolayers were formed as previously described (21, 44, 45). Briefly, 3D colonoids were washed with an ice-cold solution of 0.5 mM EDTA in PBS, and dissociated with trypsin at 37°C for 4 min. After inactivation of trypsin with 10% FBS, cells were gently pipetted up and down, and passed through a 40-mm nylon filter to produce a single-cell solution. The cell solutions were seeded onto polycarbonate Transwell filters (Corning) coated with Matrigel, incubated at 37°C for 24 h in medium containing 10 μM Y-27632 ROCK inhibitor (Sigma-Aldrich), and then grown in differentiation medium (also containing 10 μM Y-27632 ROCK inhibitor) for 4 days. Differentiation medium was changed every other day prior to infection. Confluency of the monolayers was determined by light microscopy, and polarization was verified by measuring the transepithelial electrical resistance (TEER), as previously described (27).

For infections, medium was carefully removed to avoid mechanical disruption of the mucus layer, and fresh differentiation medium was added. Colonoid monolayers were apically infected with overnight cultures at an MOI of 1 for each strain, except for *B. theta* (MOI of 10), to avoid bacterial overgrowth and cellular damage. After incubation at 37°C and 5% CO_2_ for 6 h, monolayers were washed twice with PBS, fixed with 4% paraformaldehyde/PBS for 20 min, permeabilized with Triton X-100 for 6 min, and stained with Alexa Fluor 568 phalloidin (Molecular Probes) for 30 min and DAPI for 5 min. After three washes with PBS, filters were carefully cut from the transwell insert and mounted onto glass slides with ProLong antifade. Fluorescence confocal imaging was performed using a Zeiss 880 LSM microscope.

### Scanning electron microscopy (SEM) and transmission electron microscopy (TEM).

Colonoid monolayers were grown on Transwell filters or 96-well Nunc cell culture plates (Corning) for SEM and TEM imaging, respectively. For SEM, after postinfection, samples were fixed with a solution of 2% paraformaldehyde, 2% glutaraldehyde, 1% sucrose, and 3 mM CaCl_2_ in 0.1 M sodium carbonate, postfixed with 2% osmium tetroxide, dehydrated with increased concentration of ethanol, and then subjected to critical point drying with carbon dioxide. Filters were mounted on stubs and coated with gold/palladium to be imaged on a Field-Emission Scanning Electron Microscope (Zeiss Sigma) operating at 10 kV. For TEM, samples were prepared as previously described (46). Briefly, after infection, colonoid monolayers were trypsinized, pelleted in PBS, fixed with glutaraldehyde, and then dehydrated in graded ethanol. After complete evaporation of ethanol, samples were coated with gold to be observed under TEM.

### RNA isolation for RNA sequencing.

Infections were performed on colonoid monolayers as described above. After infection, samples were immediately placed on ice and total RNA was isolated using a RNEasy minikit (Qiagen) according to the manufacturer’s instructions. The RNA quality was confirmed by using a Bioanalyzer device (RNA integrity number > 8.0). Samples were then transferred to Novogene (Sacramento, CA) for cDNA library preparation and sequencing.

### Library construction, quality control, and sequencing.

mRNA was purified from total RNA using poly-T oligo-attached magnetic beads. After fragmentation, the first strand cDNA was synthesized using random hexamer primers, followed by the second strand cDNA synthesis using either dUTP for directional library or dTTP for nondirectional library. The library was checked with Qubit and real-time PCR for quantification and bioanalyzer for size distribution detection. Quantified libraries were pooled and sequenced on Illumina platforms, according to effective library concentration and data amount.

### Data analysis.

Raw data (raw reads) of fastq format were firstly processed through in-house perl scripts, and high-quality clean data (Q20 > 98%; Q30 > 94%) was used for downstream analyses. Paired-end clean reads (range of 63–85 million per sample) were aligned to the human reference genome (hg38) using *Hisat2 v2.0.5. featureCounts v1.5.0-p3* was used to count the reads numbers mapped to each gene. Differential expression analysis of two conditions/groups (two biological replicates per condition) was performed using the *DESeq2 R* package (1.20.0). The resulting *P* values were adjusted using Benjamini and Hochberg’s approach for controlling the false discovery rate (FDR). Genes with an adjusted *P* value of ≤0.05 found by *DESeq2* were assigned as differentially expressed. Gene Ontology (GO) enrichment analysis of differentially expressed genes was implemented by the *clusterProfiler R* package, in which gene length bias was corrected. GO terms with corrected *P* value less than 0.05 were considered significantly enriched by differential expressed genes. We also used the *clusterProfiler R* package to test the statistical enrichment of differential expression genes in KEGG pathways (http://www.genome.jp/kegg/).

### Sample preparation for metabolomics.

The presence of metabolites in the extracellular media of mock/infected colonoid monolayers (exometabolome) was determined by liquid chromatography mass spectrometry (LC-MS/MS). Briefly, samples were collected after infection and passed through a 0.22-um hydrophilic PVDF filter (Millipore). For metabolites extraction, 0.5 mL of cold solvent (40:40:20 methanol:acetonitrile:water) was added to 100 μL of filtered supernatants prior to vigorous vortex and incubation at −20°C for 1 h, as previously described (47). Samples were centrifuged at 13,000 rpm for 15 min, dried by a Speedvac vacuum concentrator, reconstituted in 100 μL of mobile phase (80:20 methanol:acetonitrile), and then transferred to the UT Southwestern Medical Center Metabolomics Core.

### LC-MS/MS system.

LC-MS/MS analyses were performed on a Sciex “QTRAP 6500+” mass spectrometer equipped with an ESI ion spray source. The ESI source was used in both positive and negative ion modes. The ion spray needle voltages used for MRM positive and negative polarity modes were set at 4,800 V and −4,000 V, respectively. The mass spectrometer was coupled to Shimadzu HPLC (Nexera X2 LC-30AD). The system was under control by *Analyst 1.7.1* software.

### Chromatography conditions and data analysis.

Chromatography was performed under HILIC conditions using a SeQuant ZIC-pHILIC 5 μm polymeric 150 × 2.1 mm PEEK coated HPLC column (Millipore). The column temperature, sample injection volume, and low rate were set to 45°C, 5 μL, and 0.15 mL/min, respectively. The HPLC conditions were as follows. Solvent A: 20 mM ammonium carbonate including 0.1% ammonium hydroxide. Solvent B: Acetonitrile. Gradient condition was 0 min: 80% B; 20 min: 20% B; 20.5 min 80% B; 34 min: 80% B. Total run time: 34 min. Flow was diverted to waste for the first 1 min and after 16 min. Data were processed by SCIEX *MultiQuant 3.0.3* software with relative quantification based on the peak area of each metabolite. Metabolomic analysis was performed using *MetaboAnalyst* software (48), and metabolites with a fold change of 2 or more and FDR of <0.05 were considered as significantly changed among the samples.

### Data availability.

The processed data used for metabolomics and transcriptomics analyses are shown in Table S4 and Table S5 (at https://www.dropbox.com/scl/fi/4sc2x8k7hbi22vlm00ueq/Table-S3.xlsx?dl=0&rlkey=obe2yjtnw2k4kc2ggsgbgyigc), respectively. Table S3 shows the GO Biological Processes and KEGG Pathways significantly enriched in each condition.

10.1128/mbio.01321-22.1 TABLE S1 Strains and plasmids used in this study. Download Table S1, PDF file, 0.2 MB. Copyright © 2022 Martins et al. 2022 Martins et al. https://creativecommons.org/licenses/by/4.0/ This content is distributed under the terms of the Creative Commons Attribution 4.0 International license.

10.1128/mbio.01321-22.2 TABLE S2 Oligonucleotide primers used in this study. Download Table S2, PDF file, 0.1 MB. Copyright © 2022 Martins et al. 2022 Martins et al. https://creativecommons.org/licenses/by/4.0/ This content is distributed under the terms of the Creative Commons Attribution 4.0 International license.

10.1128/mbio.01321-22.3 TABLE S3 GO biological processes and KEGG pathways significantly enriched in each condition. Download Table S3, XLSX file, 0.09 MB. Copyright © 2022 Martins et al. 2022 Martins et al. https://creativecommons.org/licenses/by/4.0/ This content is distributed under the terms of the Creative Commons Attribution 4.0 International license.

10.1128/mbio.01321-22.4 TABLE S4 Peak area of all metabolites detected in this study. Download Table S4, XLSX file, 0.06 MB. Copyright © 2022 Martins et al. 2022 Martins et al. https://creativecommons.org/licenses/by/4.0/ This content is distributed under the terms of the Creative Commons Attribution 4.0 International license.

10.1128/mbio.01321-22.5 MOVIE S1 Dynamics of actin pedestal formation on Lifeact::GFP-expressing HeLa cells infected with EHEC alone. Download Movie S1, AVI file, 1.2 MB. Copyright © 2022 Martins et al. 2022 Martins et al. https://creativecommons.org/licenses/by/4.0/ This content is distributed under the terms of the Creative Commons Attribution 4.0 International license.

10.1128/mbio.01321-22.6 MOVIE S2 Dynamics of actin pedestal formation on Lifeact::GFP-expressing HeLa cells infected with EHEC and *B. theta*. Download Movie S2, AVI file, 0.7 MB. Copyright © 2022 Martins et al. 2022 Martins et al. https://creativecommons.org/licenses/by/4.0/ This content is distributed under the terms of the Creative Commons Attribution 4.0 International license.

10.1128/mbio.01321-22.7 MOVIE S3 Dynamics of actin pedestal formation on Lifeact::GFP-expressing HeLa cells infected with EHEC and E. faecalis. Download Movie S3, AVI file, 0.9 MB. Copyright © 2022 Martins et al. 2022 Martins et al. https://creativecommons.org/licenses/by/4.0/ This content is distributed under the terms of the Creative Commons Attribution 4.0 International license.

10.1128/mbio.01321-22.8 MOVIE S4 Dynamics of actin pedestal formation on Lifeact::GFP-expressing HeLa cells infected with EHEC and E. coli HS. Download Movie S4, AVI file, 0.9 MB. Copyright © 2022 Martins et al. 2022 Martins et al. https://creativecommons.org/licenses/by/4.0/ This content is distributed under the terms of the Creative Commons Attribution 4.0 International license.

10.1128/mbio.01321-22.9 MOVIE S5 Dynamics of actin pedestal formation on Lifeact::GFP-expressing HeLa cells infected with EHEC and a mixed culture (MC) containing all three commensal strains (*B. theta*, E. faecalis, and E. coli HS). Download Movie S5, AVI file, 0.8 MB. Copyright © 2022 Martins et al. 2022 Martins et al. https://creativecommons.org/licenses/by/4.0/ This content is distributed under the terms of the Creative Commons Attribution 4.0 International license.
