# Supplementary material for: Interactions between Enterohemorrhagic Escherichia coli (EHEC) and Gut Commensals at the Interface of Human Colonoids
Source: mBio. 2022 May 31;13(3):e01321-22. doi: 10.1128/mbio.01321-22 (PMC9239246; doi:10.1128/mbio.01321-22)
Supplement: TABLE S1 [file mbio.01321-22-s0001.pdf]

Table S1. Strains and plasmids used in this study.

Strain	Description	Reference
EHEC 86-24 (wild-type)	*E. coli* O157:H7 clinical isolate	(1)
rpsMp_Cm::LacZ	EHEC strain with engineered unique housekeeping gene. Chloramphenicol resistance gene driven by the *rpsM* promoter and cloned into the *Lac* locus	(2)
B. thetaiotaomicron VPI-5482	Human commensal strain	ATCC 29148
Bt(P_{BfP1E6}-GFP)	GFP-expressing *B. theta*	(3)
E. faecalis V583	Human blood isolate	(4)
E. coli HS	Human commensal strain	(5)
**Plasmid**	**Description**	**Reference**
pDP151	Constitutive mCherry expression. (for EHEC)	(6)
pTRKH3-ermGFP	GFP expression under control of erythromycin ribosomal methylase promoter (for *E. faecalis*)	(7)
pd2EGFP	Constitutive d2EGFP expression (for *E. coli* HS)	Clontech

1. Griffin PM, Ostroff SM, Tauxe RV, Greene KD, Wells JG, Lewis JH, Blake PA. 1988. Illnesses associated with Escherichia coli O157:H7 infections. A broad clinical spectrum. Ann Intern Med 109:705-12.
2. Cameron EA, Sperandio V, Dunny GM. 2019. Enterococcus faecalis Enhances Expression and Activity of the Enterohemorrhagic Escherichia coli Type III Secretion System. mBio 10.
3. Whitaker WR, Shepherd ES, Sonnenburg JL. 2017. Tunable Expression Tools Enable Single-Cell Strain Distinction in the Gut Microbiome. Cell 169:538-546.e12.
4. Sahm DF, Kissinger J, Gilmore MS, Murray PR, Mulder R, Solliday J, Clarke B. 1989. In vitro susceptibility studies of vancomycin-resistant Enterococcus faecalis. Antimicrob Agents Chemother 33:1588-91.
5. Levine MM, Bergquist EJ, Nalin DR, Waterman DH, Hornick RB, Young CR, Sotman S. 1978. Escherichia coli strains that cause diarrhoea but do not produce heat-labile or heat-stable enterotoxins and are non-invasive. Lancet 1:1119-22.
6. Gruber CC, Sperandio V. 2014. Posttranscriptional control of microbe-induced rearrangement of host cell actin. mBio 5:e01025-13.
7. Lizier M, Sarra PG, Cauda R, Lucchini F. 2010. Comparison of expression vectors in Lactobacillus reuteri strains. FEMS Microbiol Lett 308:8-15.
