# Supplementary material for: Interactions between Enterohemorrhagic Escherichia coli (EHEC) and Gut Commensals at the Interface of Human Colonoids
Source: mBio. 2022 May 31;13(3):e01321-22. doi: 10.1128/mbio.01321-22 (PMC9239246; doi:10.1128/mbio.01321-22)
Supplement: TABLE S2 [file mbio.01321-22-s0002.pdf]

Table S2. Oligonucleotide primers used in this study.

Primer	Sequence 5'-3'	Description
cat_RT_fwd	GTGAGCTGGTGATATGGGATAG	qRT-PCR – *cat* (endogenous control)
cat_RT_rev	CCGGAAATCGTCGTGGTATT	
ler_RT_fwd	CGACCAGGTCTGCCC	qRT-PCR – LEE1 (*ler*)
ler_RT_rev	GCGCGGAACTCATC	
escC_RT_fwd	CTGAAGACAATGGCAAGTAATGG	qRT-PCR – LEE2 (*escC*)
escC_RT_rev	ACTGCATTAAGACGTGGATCAG	
escV_RT_fwd	GAGTGCAAAAGGAAAGCCAG	qRT-PCR – LEE3 (escV)
escV_RT_rev	ATGATACCAGCAATAGCGTCC	
espA_RT_fwd	AGCTATTTGAGGAACTCGGTG	qRT-PCR – LEE4 (*espA*)
espA_RT_rev	CATCTTTTGTGCCGTGGTTG	
tir_RT_fwd	GAGGGAGTCAAATAGCGGTG	qRT-PCR – LEE5 (*tir*)
tir_RT_rev	ATCTGAACGAAGGCTGGAAG	
